# Hospitais de Manguinhos: reflexões sobre políticas científicas e patrimônio, das doenças tropicais à covid-19

**DOI:** 10.1590/S0104-59702024000100054

**Published:** 2024-10-14

**Authors:** Renato da Gama-Rosa Costa, Renata Soares da Costa Santos, Giovanna Ermida Martire

**Affiliations:** i Pesquisador e professor, chefe do Departamento de Patrimônio Histórico, Casa de Oswaldo Cruz/Fiocruz. Rio de Janeiro – RJ – Brasil renato.gamarosa@fiocruz.br; ii Pesquisadora e bolsista do Departamento de Patrimônio Histórico, Casa de Oswaldo Cruz/Fiocruz. Rio de Janeiro – RJ – Brasil rsoarescsantos@gmail.com; iii Arquiteta, Departamento de Patrimônio Histórico, Casa de Oswaldo Cruz/Fiocruz. Rio de Janeiro – RJ – Brasil giovanna.martire@fiocruz.br

**Keywords:** Arquitetura hospitalar, Covid-19, Unidades Srag

## Abstract

Em 2020 o mundo vivenciou o flagelo da covid-19. A Fiocruz se viu novamente diante de um grande desafio. Uma das estratégias estabelecidas pela instituição para o enfrentamento da doença foi a construção de um novo hospital. Não era a primeira vez que a Fiocruz construía um espaço de assistência hospitalar. O primeiro hospital, cujas instalações necessitam de ações de preservação, foi aberto em 1918 para estudos e tratamento de doenças tropicais. O segundo foi erguido em 2021, especialmente para tratar as vítimas da nova epidemia, na lógica de um hospital de campanha. Esse artigo pretende trazer reflexões para se entender o contexto de construção de ambos os hospitais, bem como contribuir para estudos sobre história das ciências e da saúde.

Os hospitais estudados^1^ neste artigo fazem parte do que hoje se constitui a Fundação Oswaldo Cruz (Fiocruz) que, ao longo de sua vida institucional, iniciada em 1900, enfrentou uma série de desafios em relação à saúde pública brasileira. Mais recentemente, por conta da covid-19, além da fabricação de vacinas, também logrou erguer um hospital para receber vítimas da doença. Não foi a primeira vez que a Fiocruz construiu um hospital em sua estrutura institucional para atender casos graves e realizar pesquisas experimentais. O primeiro foi erguido entre 1912 e 1918 para pesquisas no campo da medicina tropical; o segundo foi implantado recentemente, em 2020, para responder a uma crise sanitária deflagrada pela covid-19. Ambos são relevantes para a história institucional, e a análise de suas arquiteturas, seus contextos históricos e métodos construtivos nos possibilita pensar sob o ponto de vista da história da medicina, mas igualmente sob o ponto de vista da preservação patrimonial.

A instituição foi criada em 1900 na fazenda de Manguinhos, subúrbio do Rio de Janeiro, para produzir soro e vacina contra a peste bubônica, que acabara de ser diagnosticada entre imigrantes no porto de Santos, havendo grande temor de que alcançasse o Rio de Janeiro, capital do país na época. A direção do então Instituto Soroterápico Federal de Manguinhos foi entregue ao doutor Pedro Affonso Franco, barão de Pedro Afonso, proprietário do Instituto Vacínico Municipal, onde era produzida e aplicada a vacina antivariólica. A direção técnica coube a Oswaldo Gonçalves Cruz, jovem médico que regressara há pouco de uma temporada de estudos de especialização na França, principalmente no Instituto Pasteur de Paris. Durante a instalação do Instituto Soroterápico de Manguinhos, o prefeito da cidade do Rio de Janeiro solicitou ao Ministério da Justiça e Negócios Interiores que a instituição fosse transferida para a alçada federal, dando origem ao que hoje se identifica como Fundação Oswaldo Cruz. Em 1902, Oswaldo Cruz assumiu a direção plena do instituto e em seguida foi nomeado para o cargo de diretor-geral de Saúde Pública no governo de Rodrigues Alves, eleito presidente da República em março daquele ano, tendo como principal ponto de seu programa de governo o saneamento da capital federal.^2^

Em meio aos debates no Congresso Nacional sobre a reforma dos serviços sanitários, Oswaldo Cruz encaminhou a proposta de transformar o Soroterápico em um instituto para estudo das doenças infecciosas tropicais “segundo as linhas do Instituto Pasteur de Paris, [que] deve ser encarregado da preparação de todos os soros terapêuticos, vacinas, o tratamento antirrábico, a preparação de fermentos industriais, o ensino da bacteriologia e da parasitologia” (citado em Stepan, 1976, p.78). Buscava transformar o instituto em um núcleo de estudos experimentais capaz de acentuar “o nome de nosso país no estrangeiro” (p.78). A proposta foi aprovada em dezembro de 1907, quando já estava em curso a construção dos prédios para a instituição, rebatizada Instituto Oswaldo Cruz (IOC) em março de 1908.

Seu regulamento dava-lhe considerável autonomia administrativa e financeira, ampliava o quadro de funcionários e autorizava a venda de produtos biológicos, a prestação de serviços científicos e profiláticos a órgãos públicos e setores privados. Tais mudanças proporcionaram fecundas oportunidades aos cientistas recrutados pelo instituto para investigar doenças das cidades e do campo causadas por bactérias, protozoários e vermes, o que resultou em ações profiláticas e trabalhos científicos que levaram a instituição a ser reconhecida nacional e internacionalmente como importante centro produtor de conhecimentos nos campos da microbiologia e medicina tropical (Benchimol, 1990).

A autonomia financeira do instituto, juntamente aos conhecimentos acerca de medicina tropical, permitiu, por exemplo, que se completasse a construção do primeiro hospital aqui apresentado. Ao longo da trajetória da instituição, seu prestígio e tradição científica de atuação em diferentes campos da saúde pública possibilitaram responder a desafios sanitários com rapidez e eficiência, como a mais recente crise deflagrada pela covid-19. Assim se deu, tanto pelo lado da produção de vacinas contra a doença como pelo atendimento às suas vítimas, concretizado com a construção do Complexo Hospitalar, o segundo hospital ao qual este artigo se refere.

Atualmente, após o fim da emergência sanitária da covid-19 e a gradual desocupação dos leitos destinados exclusivamente à doença surgem questionamentos quanto à função e ao futuro de tais hospitais na instituição. O hospital centenário, com sua herança histórica e valor patrimonial, enfrenta desafios para a continuidade de sua função assistencial e de pesquisa clínica, em ambiente limitado por suas dimensões e características de hospital de pequeno porte, e pelas questões de salvaguarda cultural. Por outro lado, o novo hospital, de arquitetura emergencial, certamente exigirá ações de manutenção para continuar em funcionamento que superem as limitações da opção construtiva adotada.

## O antigo hospital de Manguinhos

O primeiro hospital aqui estudado foi idealizado por Oswaldo Cruz na primeira década do século XX, inicialmente como mais um lugar para isolamento de portadores de doenças infecciosas na cidade do Rio de Janeiro.^3^ Começou a ser construído em 1912 e passou a fazer parte do “complexo arquitetônico” em funcionamento nos terrenos da fazenda de Manguinhos, tendo como componente central o pavilhão ou castelo Mourisco.

Ele foi erguido no alto de uma colina afastada da orla marítima. Durante sua construção, a maioria dos componentes do complexo arquitetônico de Manguinhos já estava concluída, ou em fase final de acabamento. O projeto inicial para o hospital previa cinco pavilhões, com a verba pública concedida em janeiro de 1912. Mas, sem a renovação da verba inicial, esses recursos esgotaram-se no ano seguinte, resultando na entrega de apenas um pavilhão. As obras foram retomadas em 1918 com verbas próprias do instituto, obtidas, em grande parte, com a venda de uma vacina, fabricada pelo instituto, de uso veterinário contra a peste da manqueira (Benchimol, 1990, p.38).^4^

Denominado durante a construção Hospital de Moléstias Tropicais ou Hospital de Doenças Tropicais, foi inaugurado como Hospital Oswaldo Cruz em 1918. Sua inauguração se deu no momento de manifestação da epidemia de gripe espanhola que fez numerosas vítimas na cidade do Rio de Janeiro. A documentação pesquisada nos permite saber apenas que no começo de 1921 o hospital funcionava e cumpria sua missão de ser “um repositório permanente de casos clínicos que ofereçam assunto de pesquisas experimentais” (Relatório..., 1921, p.5). Em 1940, passou a ser chamado de Hospital Evandro Chagas (HEC) em homenagem ao primogênito do médico Carlos Chagas. Entre 1959 e 1978 recebeu o nome de pavilhão Gaspar Vianna. Finalmente, a partir de então, voltou a se chamar Hospital Evandro Chagas. Para contornar essa pluralidade de nomes, bem como as mudanças no estatuto da instituição à qual esteve ligado, passaremos a designar nosso objeto de estudo Hospital Evandro Chagas.

O projeto do hospital e o acompanhamento das obras ficou a cargo de Luiz Moraes Júnior (1867-1955), jovem arquiteto português contratado por Oswaldo Cruz para a realização de todas as edificações do complexo de Manguinhos, assim como de outros prédios médico-hospitalares construídos na cidade do Rio de Janeiro durante sua gestão como diretor-geral de Saúde Pública.

O hospital foi erguido em terreno afastado das demais instalações do instituto e dos bairros em formação nos arredores da fazenda de Manguinhos, o que remete à importante recomendação do paradigma da arquitetura pavilhonar, ou seja, o afastamento físico dos lugares de concentração humana para evitar infecções e contágios e para garantir a livre circulação do ar no seu entorno. Entretanto, a não construção dos demais pavilhões previstos no projeto original contrariou outra importante recomendação do sistema pavilhonar: a separação das doenças em pavilhões específicos. Os serviços foram centralizados em um único prédio, unificando-se as doenças e atividades hospitalares, o que obrigou à adoção de procedimentos pasteurianos de assepsia e antissepsia, considerados capazes de neutralizar os contágios.^5^

**Figura 1:**
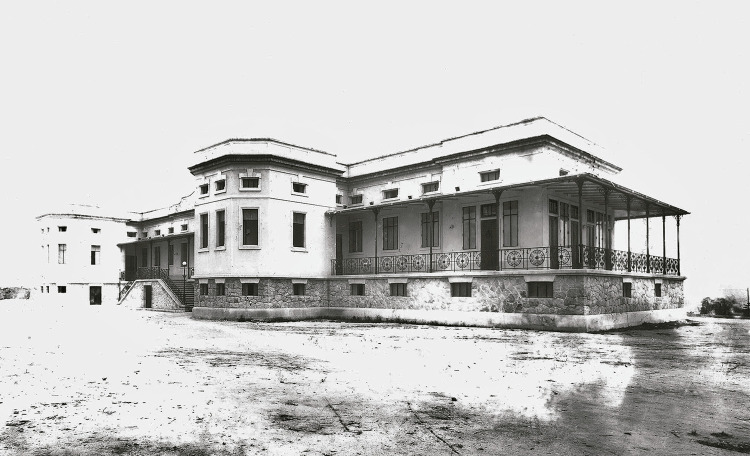
: Hospital Oswaldo Cruz, fachada principal, c.1918. Foto de J. Pinto (Departamento de Arquivo e Documentação/Casa de Oswaldo Cruz/Fiocruz)

Seu programa seguiu de perto o do hospital construído no Instituto Pasteur, de Paris. Caracterizava-se por arquitetura sóbria, limpa, com poucos elementos decorativos restritos às varandas, que proporcionavam sombra e ar às enfermarias (Figura 1). O prédio abrigava enfermarias, quartos com leitos separados por sexo, quartos para exames de raio-X e eletrocardiograma, cozinha, banheiros, lavanderia e laboratório para experimentos usando pequenos animais. Na época de sua inauguração era tido como um hospital moderno, provido de tecnologia de ponta para as pesquisas clínicas e laboratoriais, igualando-se em sofisticação tecnológica às demais unidades do IOC − possuía instalação elétrica, linha telefônica, um pequeno elevador, sistema de condicionamento de ar e equipamento médico que lhe davam importante diferencial em relação aos demais hospitais da cidade (Santos, 2019). Consta que Carlos Chagas foi o primeiro médico no país a fazer uso de um eletrocardiógrafo, para a obtenção dos traçados de ritmos cardíacos apresentados pelos portadores da tripanossomíase, descoberta em 1909, em Lassance, cidade de Minas Gerais (Kropf, 2009, p.258-259). Posteriormente, ele se utilizou de instalações do HEC para realizar pesquisas cardíacas em pacientes com diagnóstico de “moléstia de Chagas” (Santos, 2019). Um relatório institucional de atividades de 1924 menciona a “adaptação de quatro salas que serviam de banheiros para a instalação de um eletrocardiógrafo e aparelhos de raio-x” (Relatório..., 1924, p.4).

## O novo complexo hospitalar em Manguinhos

**Figura 2:**
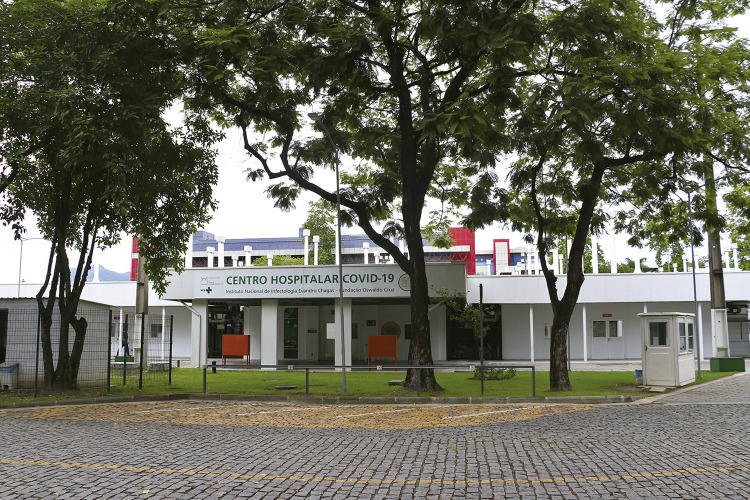
: Centro Hospitalar Covid-19, 2023. Foto de Peter Iliciev (Coordenação de Comunicação Social/Fiocruz)

O Centro Hospitalar Covid-19, do Instituto Nacional de Infectologia (INI/Fiocruz) (Figura 2), foi construído em sessenta dias, em caráter emergencial, como continuidade às ações de enfrentamento à pandemia de covid-19 pela Fiocruz. O empreendimento foi uma importante estratégia da Fiocruz no combate à pandemia no Brasil, conjugando assistência e estudos clínicos, com leitos exclusivos de tratamento intensivo e semi-intensivo de pacientes graves infectados pela covid-19.

O complexo hospitalar foi erguido em área de 9.850 metros quadrados, próximo a uma das entradas do *campus* Manguinhos pela avenida Brasil, em local onde funcionava um campo de futebol da associação de servidores da instituição, utilizado por funcionários em momento de lazer. A empresa responsável pela construção foi a RAC Engenharia S/A.^6^ O engenheiro responsável pelo projeto foi Carlos German, e a equipe de arquitetos foi composta por profissionais com especialidades diversas, como arquitetura hospitalar, arquitetura de interiores, urbanismo e gerência administrativa. À frente do projeto esteve a arquiteta Vânia Furugem, com os aconselhamentos do projeto hospitalar, visto sua maior intimidade com a arquitetura hospitalar, devido a especializações acadêmicas e a interesses e trabalhos realizados anteriormente (Costa et al., 28 jan. 2022).

As obras foram iniciadas em 5 de abril de 2020, e a partir de 19 de maio de 2020 o hospital foi aberto à Central de Regulação do Estado do Rio de Janeiro, passando a receber, gradativamente, seus primeiros pacientes para internação. Concluído no mês em que a pandemia atingiu um pico de infecção, justamente pelo alto índice de infecção, a expectativa era grande para sua inauguração e seu funcionamento. Além disso, circulavam notícias sobre a demora no prazo para entrega dos hospitais de campanha, medida de urgência para conter o crescente número de mortes registrado diariamente no país. Uma reportagem veiculada em maio de 2020 chamava a atenção para o fato de que, dos 1.300 leitos prometidos pelos hospitais de campanha, apenas duzentos, ou seja, os disponibilizados pela Fiocruz, haviam sido entregues e estavam em funcionamento (Centro hospitalar..., 20 maio 2020).

A unidade hospitalar de atendimento e pesquisa foi construída com a proposta de tecnologia de “salas limpas” e com características que a diferenciam das unidades de campanha erguidas pelo país nesse mesmo contexto. Enquanto a maioria dos projetos hospitalares foi estruturada para funcionamento temporário, os chamados hospitais de campanha, o Centro Hospitalar Covid-19 da Fiocruz foi projetado desde o início com o compromisso de permanecer aberto após a pandemia e implantado como um legado da Fiocruz para o Sistema Único de Saúde (Fiocruz, 19 maio 2020; Azevedo, 4 out. 2020).

Embora apresente a peculiaridade de almejar ser um hospital permanente, comparando-o aos hospitais temporários também projetados na pandemia de covid-19 no país, há semelhanças indiscutíveis, justificadas pela velocidade para execução do projeto. No Brasil, a maioria dos hospitais temporários foi construída por meio do sistema tênsil, considerado o mais característico ao tratar de arquitetura efêmera emergencial. Sobre o modelo, destaca-se uma estrutura “formada por peças rígidas em metal, madeira ou até plástico com rápida montagem e sistema flexível”, comuns em eventos e feiras, “adaptadas a este fim em um emaranhado labiríntico de tendas em lona antichama, divisórias e pisos elevados” (Ghisleni, 31 out. 2021).

A questão da adaptabilidade dos espaços e dos materiais usados foi central para a criação dos hospitais temporários pensados como resposta à crise sanitária, somando-se a esse aspecto a preocupação com a eficiência em conter a transmissão do vírus nos espaços (Ruprecht, 1 abr. 2020).

Outro ponto importante dessas construções temporárias era o fato de estar destinadas a desafogar o sistema de saúde, conduzindo a tais unidades aqueles pacientes menos comprometidos pela doença. De acordo com Fabio Racy, especialista em medicina do desastre e coordenador médico do hospital de campanha do Pacaembu, gerenciado pelo Hospital Israelita Albert Einstein, a pandemia de covid-19 exigia muitas internações, saturando o sistema de saúde e, portanto, “os hospitais de campanha ajudam principalmente a desafogar a demanda por leitos para pacientes com covid-19 de baixa complexidade” (citado em Ruprecht, 1 abr. 2020).

A nova instalação hospitalar do *campus* de Manguinhos foi adequada para atender às normas mais atuais relacionadas à área de saúde e contou com processo construtivo semelhante, em certa medida, ao modelo implantado de arquitetura hospitalar temporária característico da conjuntura em que fora erguido, lançando dúvida sobre sua vida útil estendida. Sua construção no contexto da emergência dotou-o de elementos de um típico hospital de campanha, quais sejam, modulação, rápida montagem e instalações padronizadas. A semelhança com hospitais construídos para a pandemia de covid-19, incluindo exemplos em outros países^7^ e com parâmetros prescritos pela própria Organização Mundial de Saúde (OMS) para a construção de unidades de tratamento de Síndrome Respiratória Aguda Grave (Srag), não esconde a opção adotada nos parâmetros do projeto de arquitetura realizado pela Fiocruz para as novas instalações hospitalares. Nas premissas da OMS, aparecem, com clareza, as diretrizes apresentadas no curso oferecido pela entidade para arquitetos e engenheiros do ramo: “O pacote de treinamento ‘Unidades para SRAG’ foi desenvolvido para atender às necessidades operacionais que surgiram com a pandemia covid-19” (WHO, s.d.). De fato, uma análise do projeto nos permite perceber que o novo hospital erguido em Manguinhos seguiu as premissas apresentadas no curso da OMS, confirmando seu caráter emergencial e colocando dúvidas sobre sua permanência sem um alto investimento para manutenção estrutural.

O Centro Hospitalar Covid-19 foi concluído com o total de 195 leitos, todos em quartos individuais intensivos e semi-intensivos com isolamento dos pacientes. O projeto reúne o que há de mais moderno em sua tecnologia e estrutura. Todos os leitos operam com um sistema de isolamento com pressão negativa do ar, específico para infecções por aerossóis. No interior dos quartos individuais, uma tubulação é responsável por sugar o ar contaminado que passa por um sistema de filtragem antes de ser eliminado por chaminés instaladas na parte externa da construção.

O complexo hospitalar conta com uma central de tratamento de esgoto própria, concebida para tratar resíduos do coronavírus e garantir destino seguro do efluente gerado. Dos leitos, todos possuem assistência de alta complexidade e sistema de apoio diagnóstico próprio, o que inclui aparelhos de raio-x, ultrassonografia, ecocardiografia, tomografia computadorizada e serviços de broncoscopia e endoscopia, patologia clínica e agência transfusional. A unidade é autossuficiente – tem fornecimento de energia, geradores e reservatórios de água e toda a infraestrutura exigida para um hospital desse porte, independente das demais áreas da Fiocruz no *campus*. Também conta com uma entrada exclusiva para ambulâncias e heliponto, tem uma área de apoio técnico, com farmácia, nutrição e Central de Material Esterilizado. Referente ao apoio administrativo, conta com espaço para gestão, acolhimento, assistência à família e *call center*. E, de apoio logístico, o projeto possui segurança, hotelaria, refeitório, almoxarifado e necrotério.

Do ponto de vista da assistência e pesquisa sobre pacientes, o Centro Hospitalar Covid-19 apresenta uma característica que conecta diferentes temporalidades da história institucional. O espaço hospitalar integra o Instituto Nacional de Infectologia Evandro Chagas (INI/Fiocruz) e mantém o vínculo entre pesquisas clínicas e terapêuticas com atendimento a pacientes, desde o início do século XX (Santos, 2019). O novo centro de atenção e pesquisa foi considerado peça fundamental para acelerar os estudos conduzidos pelo INI e toda a rede de colaboração da Fiocruz no Brasil e internacionalmente. De acordo com Valdilea Veloso, diretora do INI, um exemplo é o ensaio clínico Solidarity, da OMS, que pesquisa os medicamentos usados para o tratamento da covid-19 (WHO, 2021).

O estudo investiga a eficácia de quatro desses medicamentos no tratamento do novo coronavírus, dele participando, além do centro hospitalar do INI/Fiocruz, outros 17 hospitais de 12 estados brasileiros. “Coordenado no Brasil pela Fiocruz, o Solidarity é um ensaio clínico randomizado e adaptativo, permitindo que, com o surgimento de novas evidências científicas ao longo do estudo, haja alteração das propostas terapêuticas” (Fiocruz, 2020). Tais relações evidenciam a presença da instituição, alinhada a esforço mundial, para dar respostas rápidas sobre quais medicamentos são eficazes no tratamento da covid-19 e quais são ineficazes e não devem ser utilizados. Demonstram, também, o comprometimento institucional em nome da saúde pública, o que fica claro no discurso da então presidenta da fundação, Nísia Trindade Lima, durante visita de reconhecimento ao local após a entrega do projeto hospitalar, em que enaltece o papel da Fiocruz no enfrentamento da doença, o legado do novo hospital e sua importante contribuição para o Sistema Único de Saúde (SUS) brasileiro:

Neste momento, em que acompanhamos com tanta preocupação o aumento de casos e de mortes em nosso país, e em particular no Rio de Janeiro, é com grande emoção que entregamos esse hospital dedicado exclusivamente à covid-19 e que permanecerá como um legado para o Sistema Único de Saúde. A Fiocruz continuará trabalhando incessantemente para fortalecer as ações do SUS em meio a esta crise humanitária que tem tido impacto tão grande na vida da população e que traz tantos desafios para um país continental e desigual como o Brasil (Fiocruz, 19 maio 2020).

## Discursos de modernidade e permanência dos hospitais de Manguinhos

A construção desses dois hospitais, separados por mais de 100 anos, nos permite analisar sob o ponto de vista da história das ciências, mas igualmente sob o ponto de vista da história da arquitetura hospitalar, as estratégias e o contexto em que cada um foi concebido. Ambos foram construídos dentro de uma política institucional de aliar tratamento à pesquisa clínica e terapêutica, ampliando, assim, um olhar mais atencioso e mais permanente à questão, evitando uma atitude meramente emergencial para o enfrentamento de pandemias. Ambos se cercaram das mais modernas tecnologias então existentes. Suas arquiteturas, entretanto, revelam atitudes específicas para cada projeto.

O primeiro hospital foi concebido dentro de um plano mais ambicioso, que teve de ser adaptado em função de situações alheias ao seu desenvolvimento, como a morte prematura do seu idealizador. Fruto de um processo em que se experimentaram diferentes concepções e estruturas formais, envolvendo pelo menos dois ou três projetos, o então Hospital Oswaldo Cruz perseguiu a tipologia de um hospital pavilhonar e de isolamento, tendo como inspiração estruturas desenvolvidas a partir dos estudos de Florence Nightingale e de Pasteur, como vimos. Segundo Nightingale (1863, p.56):

O primeiro princípio da construção hospitalar é o de internar os doentes em pavilhões separados. Por hospital pavilhonar se entende um edifício isolado, capaz de conter o maior número de leitos que um espaço pode abrigar em segurança, contendo copas e quartos de enfermeiras, lavatórios, latrinas e banheiros completos e adequados, proporcionais ao número de leitos para doentes, devidamente distantes de outros pavilhões existentes ou mesmo dos escritórios administrativos, mas interligados por corredores abertos e iluminados. Um pavilhão é, de fato, um hospital isolado, que possui, ou deveria possuir, uma pequena conexão arejada e ventilada com o restante do hospital, mas como se estivesse verdadeiramente situado a milhas de distância.^8^

Para tanto, esse hospital se estruturou em pavimento térreo, mais porão e sótão. No andar principal, distribuiu enfermarias nas extremidades do espaço, intercalando-as com locais para recepção e atendimento, e para abrigo dos médicos e enfermeiras. Tanto o porão quanto o sótão se revelam como áreas de apoio e de espaço técnico, para instalação de elementos que contribuíssem para a aeração das enfermarias. Da mesma forma, as varandas externas diminuem a incidência do sol e contribuem para amenizar o forte calor dos trópicos. Sua localização obedeceu ao distanciamento e o isolamento dos demais edifícios componentes da instituição, bem como de demais áreas da própria cidade do Rio de Janeiro. A sua volta, foram construídas instalações de suporte operacional, como lavanderia, cozinha, biotério e ambulatórios.

Sua permanência no *campus*, após mais de 100 anos de sua construção e a despeito de algumas tentativas de substituição e esvaziamento, revela sua importância histórica e patrimonial, e, portanto, interesse de preservação. A chegada do novo hospital, construído no contexto da pandemia de covid-19, alertou-nos sobre sua continuação como hospital e sua consequente salvaguarda.

O moderno complexo hospitalar de saúde do antigo IOC se efetivou na construção de um dos seis pavilhões idealizados por Oswaldo Cruz (Benchimol, 1990), como vimos. Essa edificação concebida como parte dessa grande estrutura para atendimento de pacientes separados por doenças em pavilhões centralizou todas as ações de tratamento e pesquisas das mais diversas doenças, tendo assim, já em sua origem, excedido o uso e a ocupação para a qual foi construída. Compreende-se então que o antigo hospital vem sofrendo adaptações desde o início de sua existência, seja para ocupação dos espaços, seja para modernização das instalações, de forma a garantir o cumprimento de normas, o melhor atendimento de pacientes e a realização de pesquisas.

Atualmente tem sido bastante discutida a questão da preservação da antiga edificação hospitalar em face da manutenção do uso original, tendo em vista as características arquitetônicas que a identificam como importante patrimônio da saúde. As análises que têm sido feitas ao longo dos anos certificam que a edificação se mantém preservada em sua volumetria. Alguns aspectos importantes, contudo, foram perdidos nos espaços internos, como nas divisões entre os leitos, instalações e no sistema de troca de ar e climatização originais, tanto quanto na ambiência externa e nos elementos construtivos, em especial na cobertura do telhado, nos gradis de fechamento das varandas, esquadrias e acessos, entre outros. Essas perdas comprometeram de forma quase irreversível algumas das particularidades mais importantes da tipologia de arquitetura pavilhonar, como a renovação do ar das enfermarias e o distanciamento de outras construções.

Por outro lado, sendo o primeiro hospital da Fundação Oswaldo Cruz, existe um apelo em relação a seu uso original e ao fato de que a perda desse *status* possibilitaria a redução de seu valor como monumento. Se, no entanto, a tal impossibilidade de preservação for verdadeira, seu uso original é de fato o valor mais importante a ser discutido? Diante das perdas, das dificuldades de projetos de adaptações, instalações e até mesmo de serviços simples de conservação, é preciso analisar a questão de forma mais criteriosa, levando em conta que, embora o uso original seja importante, a edificação em si é ainda mais importante e relevante, pois é somente com suas características materiais preservadas que é possível agregar valores e reconhecer sua antiguidade.

O uso original é uma qualidade especial dessa antiga edificação, reconhecido como um dos seus valores, que foram atribuídos no documento de *Política de preservação e gestão de acervos culturais das ciências e da saúde da Casa de Oswaldo Cruz* (Fiocruz, 2013), unidade da Fiocruz responsável pela sua preservação. E, neste momento, o antigo hospital está em processo de reconhecimento como patrimônio pelo Instituto de Patrimônio Histórico e Artístico Nacional (Iphan). Nos últimos anos, especialistas do Iphan têm realizado visitas para analisar e verificar se as alterações sofridas pelo edifício alteraram as características fundamentais de sua arquitetura, a ponto de comprometer sua proteção patrimonial pelo órgão: “Uma futura salvaguarda por parte do Iphan será um passo importante a se agregar às ações de valorização deste patrimônio da saúde” (Costa, Martire, 2023, p.70).

Do ponto de vista exclusivamente arquitetônico, não há expectativas de preservação de seus atributos originais, em função das necessárias e recorrentes adaptações do hospital. E, mesmo tratando-se de edificação considerada patrimônio arquitetônico, é preciso analisar o tema hospitalar por seu lado mais contemporâneo, já que sua principal função é o atendimento de pessoas doentes, bem como o desenvolvimento de pesquisas.

Nesse ponto, a construção de um novo hospital sob a responsabilidade do INI vem auxiliar nesse esforço das equipes responsáveis pela antiga edificação em adaptar a arquitetura histórica à contemporaneidade de sua atividade como hospital, com o desafio de respeitar a preservação de sua arquitetura. É necessário ter em mente que essa mudança de atividades clínicas para o novo hospital pode trazer ressignificações para o antigo hospital, mas não pode abrir espaço para seu abandono. As análises criteriosas deverão então se voltar para a questão patrimonial, pretendendo reuso que revalorize a arquitetura e favoreça a preservação da arquitetura do antigo hospital sob aspectos de rememoração da história da saúde.

Por sua vez, as novas instalações hospitalares representaram a oportunidade de a instituição ter um hospital adequado para atender às normas mais atuais relacionadas à área de saúde, embora o processo construtivo do novo hospital não permita vida útil muito longa, segundo a arquiteta que acompanhou a obra. Sabemos que ele foi construído no contexto da emergência e adotou elementos de um típico hospital de campanha, quais sejam, modulação, rápida montagem e instalações padronizadas.

O INI não reconhece que o novo complexo hospitalar tenha prazo de validade para seu funcionamento, outra característica dos hospitais de campanha. Segundo reportagem de 20 de maio de 2020, vinculada na edição de meio-dia do *Jornal Hoje* (Rede Globo de Televisão), a instituição afirmou que “o Centro Hospitalar Covid-19 não é um hospital de campanha e que ‘vai permanecer de pé após a pandemia’” (Centro hospitalar..., 20 maio 2020). O arquiteto Luiz Carlos Toledo (29 jun. 2020), em seu artigo sobre os hospitais construídos no âmbito do combate à covid-19, no Rio de Janeiro, também chegou a afirmar que, no caso da Fiocruz, seu hospital teria sido construído como “permanente, integrado ao Sistema Único de Saúde”. O arquiteto chama a atenção para o equívoco de se erguer hospitais de campanha para tal fim, pois, segundo ele, ainda “terão que funcionar por um longo tempo”, em razão da permanência da pandemia, e porque a estrutura de tais hospitais não seria a mais apropriada para a situação.

Além disso, Toledo (29 jun. 2020) reconhece que o exemplo trazido pelo novo hospital da Fiocruz chamou a atenção para alguns aspectos importantes para seu futuro: “195 leitos de tratamento intensivo e semi-intensivo, servidos por um sistema de exaustão e filtragem do ar para diminuir o risco da transmissão da covid-19 no próprio ambiente hospitalar, protegendo as equipes de saúde e os pacientes de maior exposição ao vírus”. Outros aspectos poderão ser aprimorados, como a criação de Unidades de Tratamento Intensivo individuais, sistemas mais eficientes de troca do ar interno, mais espaço para os leitos e mais atenção aos pacientes, com aprimoramento das tecnologias de comunicação entre médicos, pacientes e seus familiares, além da recuperação de elementos tradicionais dos antigos hospitais, como mecanismos naturais de ventilação e insolação, tão negligenciados nos hospitais contemporâneos.

Como vimos, as propostas para os dois hospitais, ambos construídos para emergências, separados por mais de 100 anos de construção, revelam atitudes projetuais distintas e, consequentemente, requerem formas de preservação distintas. O novo hospital se assemelha ao antigo posto que foi erguido diante de forte apelo por respostas no campo científico para conformar uma doença e conter seu avanço. Dessa vez o argumento estava centrado na pandemia de covid-19, motivando estudos e pesquisas em escala internacional para a configuração do quadro clínico, terapêutico e profilático da doença.

## Considerações finais

Na perspectiva da história da saúde e das doenças, o estopim para a materialização do antigo e do novo hospital foram as epidemias da doença de Chagas e a de covid-19.^9^ Apesar de peculiaridades históricas, etiologia, grau de contaminação e letalidade, e de todo contexto sociocultural que envolve a conformação das doenças, ambas estavam na agenda política do país e da instituição que hoje é a Fiocruz. A história dessas doenças percorreu contextos de inquietações científicas em momentos de dúvidas sobre como proceder no desenho das novas moléstias. Foram momentos de incerteza diante dos sinais e evolução dos quadros clínicos e temor social em relação às mortes em números crescentes. E, nesse contexto, respostas científicas circularam por esses hospitais e contribuíram, dentro de uma rede ampla de estudos, para definir, conter, tratar e prevenir o surto de infecções, tanto no início do século XX quanto recentemente, no início do século XXI. Nos dois momentos as pesquisas estiveram diretamente alinhadas aos estudos realizados em escala global e tiveram um espaço para experimentação hospitalar em Manguinhos.

O antigo hospital, o centenário Hospital Oswaldo Cruz, impõe a necessidade de suscitar constantes reflexões sobre a preservação de seu espaço e de sua memória. O novo hospital, o Centro Hospitalar Covid-19, coloca-nos diante de uma problemática de “nosso próprio tempo”.^10^ Sua interpretação expõe questões latentes do tempo presente, apresentando a dificuldade de experienciar e estudar, a um só tempo, os fatos históricos e a contemporaneidade.

Mas, como objeto de análise, amplia debates que circundam tanto a crise sanitária quanto os desafios à manutenção do novo hospital dessa instituição. As questões levantadas por ambos os hospitais permitem manter atualizados possíveis leituras e testemunhos das formas de fazer políticas de saúde e de conduzir cuidados médicos. Nos dois casos, foram desenvolvidos pesquisas, protocolos clínicos e terapêuticos que se mostraram cruciais para o enfrentamento das moléstias de suas respectivas épocas.

Preservar ou analisar suas formas construtivas e os contextos em que ambos foram erguidos ajuda a instituição a pensar estratégias para sua manutenção, seja pelo viés patrimonial e de valorização de sua memória, seja pela importância da atuação assistencial necessária, não mais pela urgência da pandemia, mas pela necessidade de dar prosseguimento aos estudos sobre doenças, como a covid-19, e pela carência de hospitais na cidade e na região. Nesse sentido, o artigo aqui apresentado buscou trazer reflexões que possam contribuir para entender melhor ambos os hospitais e, assim, integrar estudos sobre a história das ciências e da saúde.

## NOTAS

1 Este artigo apresenta parte das reflexões surgidas na pesquisa “Lugares para a ciência: evolução dos *campi* da Fiocruz em Manguinhos e Mata Atlântica”, com financiamento do Programa de Incentivo ao Desenvolvimento Institucional (Pidi), da Casa de Oswaldo Cruz/Fiocruz.

2 Para a história da instituição, ver Aragão (1950); Stepan (1976); Benchimol (1990); Benchimol, Teixeira (1993); Cukierman (2007).

3 Sobre o projeto e construção do hospital, ver Benchimol (1990); Santos (2019).

4 O regulamento aprovado para o IOC em 19 de março de 1908 sancionou a venda de produtos biológicos, como soros e vacinas. Essa prerrogativa foi responsável “por sua capacidade de sobreviver à penúria de recursos orçamentários votados pelo Congresso, em condições mais favoráveis do que outros estabelecimentos públicos do país” (Benchimol, 1990, p.38).

5 Análise da arquitetura do hospital encontra-se em Benchimol (1990, v.2, p.39-54); Costa (2011) e Santos (2019). Sobre a arquitetura hospitalar das primeiras décadas do século XX na cidade do Rio de Janeiro, ver Costa (2011); Sanglard (2008); Porto (2008).

6 Processo n.25389.000027/2020-78 e contrato da licitação n.11/2020.

7 Para conhecimento de outros hospitais construídos pelo mundo para a pandemia de covid-19, ver Bittencourt, Vilas-Boas, Silva (2021).

8 Nessa e nas demais citações de textos publicados em outros idiomas, a tradução é livre.

9 No artigo, tratamos as doenças não apenas como entidades biológicas e físicas, mas como fenômenos sociais e culturais, como problematizados por Cunningham, Williams (1992); Rosenberg, Golden (1997).

10 A história do tempo presente, como campo de estudos, nos conduz a reflexões sobre a escrita da história concomitante a nossa existência. Nos aproxima dos limites e desafios sobre a prática narrativa situada em “nosso próprio tempo”, conceito explorado pela historiografia nas duas últimas décadas. Sobre o tempo presente, ver Dosse (2009); Ferreira (2018, 2000).
