# Supplementary material for: Hospitais de Manguinhos: reflexões sobre políticas científicas e patrimônio, das doenças tropicais à covid-19
Source: Hist Cienc Saude Manguinhos. 2024 Oct 14;31(Suppl 1):e2024054. [Article in Portuguese] doi: 10.1590/S0104-59702024000100054 (PMC11472717; doi:10.1590/S0104-59702024000100054)
Supplement: Translation

# Translation: Manguinhos hospitals: reflections on scientific politics and heritage, from tropical diseases to covid-19

**DOI:** 10.1590/S0104-59702024000100054en

**Authors:** Renato da Gama-Rosa Costa, Renata Soares da Costa Santos, Giovanna Ermida Martire

**Affiliations:** i Researcher and teacher, chief architect, Departamento de Patrimônio Histórico, Casa de Oswaldo Cruz/Fiocruz.Rio de Janeiro – RJ – Brasil; renato.gamarosa@fiocruz.br; ii Researcher, Departamento de Patrimônio Histórico, Casa de Oswaldo Cruz/Fiocruz. Rio de Janeiro – RJ – Brasil; rsoarescsantos@gmail.com; iii Architect, Departamento de Patrimônio Histórico, Casa de Oswaldo Cruz/Fiocruz.Rio de Janeiro – RJ – Brasil; giovanna.martire@fiocruz.br

**Keywords:** Hospital architecture, Covid-19, SARI units

## Abstract

In 2020 the world suffered from covid-19. Fundação Oswaldo Cruz faced itself again with a challenging task. One of its strategies while battling the disease was building a hospital. It was not the first time the institution would build such a healthcare facility. The first hospital, which nowadays needs preservation measures, opened in 1918 intending to study and treat tropical diseases. The second, built in 2021, was a field hospital designed to treat the new epidemic victims. This article aims to reflect upon both hospitals’ construction contexts, as well as contribute to studies on the history of science and health.

The hospitals studied^1^ in this paper are a part of what today constitutes the Oswaldo Cruz Foundation (Fundação Oswaldo Cruz, Fiocruz), which throughout its life, started in 1900, faced a series of challenges regarding Brazilian public health. More recently, due to covid-19, besides producing vaccines, Fiocruz also managed to build a hospital designed to treat the disease’s victims. This was not the first time Fiocruz built a hospital within its campus intended to treat grave cases and perform experimental research. The first was built between 1912 and 1918 to study tropical diseases; the second was implemented recently, in 2020, to respond to the sanitation crisis put forth by covid-19. Both hospitals are relevant to the institution’s history and the architectural analyses, their historical contexts, and constructive methods allow us to ponder about medicine history, as well as the buildings’ preservation.

Fiocruz was created in 1900 on the Manguinhos farm, on the outskirts of Rio de Janeiro, to produce serum and vaccine against the bubonic plague, a disease recently diagnosed amongst immigrants in the port of Santos, and there was great fear it would reach Rio de Janeiro, the country’s capital at the time. The management of the then Manguinhos Federal Serotherapeutic Institute (Instituto Soroterápico Federal de Manguinhos) was handed over to Dr. Pedro Affonso Franco, Baron of Pedro Afonso, owner of the Municipal Vaccination Center (Instituto Vacínico Municipal), where the smallpox vaccine was produced and applied. The technical director was Oswaldo Gonçalves Cruz, a young doctor who had recently returned from specialized studies in France, mostly at the Pasteur Institute in Paris. During the establishment of the Manguinhos Federal Serotherapeutic Institute, the mayor of the city of Rio de Janeiro asked the Ministry of Justice and Internal Affairs to transfer the institution to federal jurisdiction, giving rise to the Manguinhos Federal Serotherapeutic Institute, today the Oswaldo Cruz Foundation. In 1902, Oswaldo Cruz took over the full management of the institute and was then promoted to Director-General of Public Health in the government of Rodrigues Alves, who had been elected President of the Republic in March of that year, with the sanitation of the federal capital as the focus of his government program.^2^

Amidst the National Congress debates on sanitation services reformation, Oswaldo Cruz proposed transforming the Manguinhos Federal Serotherapeutic Institute into an organization to study infectious tropical diseases “along the lines of the Pasteur Institute in Paris, [which] should be in charge of the preparation of all therapeutic sera, vaccines, anti-rabies treatment, the preparation of industrial ferments, the teaching of bacteriology and parasitology” (cited in Stepan, 1976, p.78). Cruz sought to transform the institute in a center for experimental studies capable of accentuating “the name of our country abroad” (p.78). The proposal was approved in December 1907, when the construction of buildings for the institution, renamed the Oswaldo Cruz Institute (Instituto Oswaldo Cruz, IOC) in March 1908, was already underway.

The Oswaldo Cruz Institute’s regulation gave it considerable administrative and financial autonomy, expanded its staff, and authorized the sale of biological products and the provision of scientific and prophylactic services to public bodies and private sectors. Such changes provided fruitful opportunities for scientists recruited by the institute to investigate city and countryside diseases caused by bacteria, protozoa, and worms, which resulted in prophylactic measures and scientific work that led the institution to be nationally and internationally recognized as an important knowledge-producing center in the fields of microbiology and tropical medicine (Benchimol, 1990).

The institute’s financial autonomy, together with its knowledge of tropical medicine, made it possible, for example, to complete the construction of the first hospital presented here. Throughout the institution’s history, its prestige and scientific tradition of working in different fields of public health have made it possible to respond quickly and efficiently to health challenges, such as the most recent crisis triggered by covid-19. This has been the case both in terms of producing vaccines against the disease and in terms of providing care for its victims, which was achieved with the construction of the Hospital Complex, the second hospital to which this article refers.

Nowadays, after the covid-19 sanitation emergency and the gradual vacating of beds dedicated exclusively to the disease, questions are being asked about the role and the future of such hospitals in the institution. The centenary hospital, with all its historic inheritance and patrimonial value, faces challenges in continuing to provide care and clinical research, in an environment limited by its dimensions and characteristics as a small hospital, and by cultural safeguarding issues. On the other hand, the new hospital, of emergency architecture, will certainly require maintenance actions to keep operating and overcome the limitations of its construction method.

## The old Manguinhos hospital

The first hospital studied here was idealized by Oswaldo Cruz in 1910’s, initially as another place to isolate people with infectious diseases in the city of Rio de Janeiro.^3^ The hospital began to be built in 1912 and it became a part of the “architectural complex” in operation on the grounds of the Manguinhos estate, with the Moorish Pavilion, or Moorish Castle, as its central component.

The hospital was built atop a hill far from the coast. During its construction, most of the buildings from the Manguinhos architectural complex were already finished, or in the final stages of completion. The original project for the hospital envisaged five pavilions, with funds granted in January 1912. However, without the initial funding being renewed for the following year, these resources ran out, resulting in the erection of only one pavilion. Construction works were resumed in 1918 with the institute’s funds, largely obtained from the sale of its vaccine for veterinary use against the mange plague (Benchimol, 1990, p.38).^4^

Named Tropical Ailments Hospital (Hospital de Moléstias Tropicais) or Tropical Diseases Hospital (Hospital de Doenças Tropicais) during its construction, the hospital was inaugurated as Oswaldo Cruz Hospital (Hospital Oswaldo Cruz) in 1918. It was inaugurated at the height of the Spanish flu epidemic, which claimed many victims in Rio de Janeiro. The documentation collected by our research only lets us know that at the beginning of 1921, the hospital was functioning and fulfilling its mission of being “a permanent repository of clinical cases that offers subjects for experimental research” (Relatório…, 1921, p.5). In 1940, it was renamed as Evandro Chagas Hospital (Hospital Evandro Chagas, HEC), in honor of Doctor Carlos Chagas’s first-born son. Between 1959 and 1978, the hospital was called Gaspar Vianna Pavilion. Finally, it was renamed Evandro Chagas Hospital once more. To get around this plurality of names, as well as the changes in the statute of the institution to which it was linked, we will now call our object of study the Evandro Chagas Hospital.

Luiz Moraes Júnior (1867-1955), a young Portuguese architect hired by Oswaldo Cruz to design all the buildings in the Manguinhos complex, as well as other medical-hospital buildings constructed in the city of Rio de Janeiro during his tenure as director-general of Public Health, designed the hospital and oversaw its construction.

The hospital was built on a plot far from the institute’s other facilities and from the neighborhoods that were being formed around the Manguinhos farm, which goes back to the important recommendation of the pavilion architecture paradigm, i.e. the physical distance from places of human concentration to avoid infection and contagion, and to allow for the free circulation of air in its surroundings. However, the failure to build the other five pavilions foreseen in the original project went against another important recommendation of the pavilion system: the separation of diseases into different pavilions. Services were centralized in a single building, unifying diseases and hospital activities, which meant that Pasteurian asepsis and antisepsis procedures had to be adopted, in the belief that they could neutralize contagions.^5^

The hospital’s program closely followed that of the hospital built at the Pasteur Institute in Paris. It was characterized by a sober, clean architecture, with a few decorative elements restricted to the balconies, which provided shade and air to the wards (Figure 1). The building housed infirmaries, rooms with beds separated by gender, rooms for x-ray and electrocardiogram examinations, a kitchen, bathrooms, laundry, and a laboratory for small animals experiments. At the time of its inauguration, it was considered a modern hospital, equipped with state-of-the-art technology for clinical and laboratory research, equal in technological sophistication to the other IOC units – it had electricity, a telephone line, a small elevator, an air conditioning system, and medical equipment that set it apart from other hospitals in the city (Santos, 2019). It is said that Carlos Chagas was the first doctor in the country to use an electrocardiograph to obtain heart rhythm tracings from patients with trypanosomiasis, discovered in 1909 in Lassance, a town in Minas Gerais (Kropf, 2009, p.258-259). He later used the HEC’s facilities to carry out cardiac research on patients diagnosed with “Chagas ailment” (Santos, 2019). An institutional activity report from 1924 mentions the “adaptation of four rooms that served as bathrooms for the installation of an electrocardiograph and x-ray machines” (Relatório…, 1924, p.4).

**Figure 1:** : Oswaldo Cruz Hospital, main façade, c.1918. Photo by J. Pinto (Departamento de Arquivo e Documentação/Casa de Oswaldo Cruz/Fiocruz)

## The new hospital complex in Manguinhos

The National Institute of Infectology’s (Instituto Nacional de Infectologia, INI/Fiocruz) (National Institute of Infectology) Covid-19 Hospital Center (Figure 2) was built in sixty days, on an emergency basis, as a continuation of Fiocruz’s actions to combat the covid-19 pandemic. The enterprise was an important strategy for Fiocruz in the fight against the pandemic in Brazil, combining care and clinical studies, with exclusive beds for intensive and semi-intensive treatment of the critically ill patients infected with covid-19.

**Figure 2:** : Covid-19 Hospital Center, 2023. Photo by Peter Iliciev (Coordenação de Comunicação Social/Fiocruz)

The hospital complex was built in a 9,850m^2^ area, next to one of the entrances to the Manguinhos campus at Brasil Avenue, on the site of a soccer pitch belonging to the institution’s staff association, used by employees for leisure purposes. RAC Engenharia S/A was the company responsible for the construction of the hospital.^6^ The engineer in charge of the project was Carlos German, and the team of architects was made up of professionals with different specialties, such as hospital architecture, interior design, urban planning, and project management. Leading the project was architect Vânia Furugem, who advised the hospital project, given her great familiarity with hospital architecture, due to her academic specializations and previous works (Costa et al., 28 jan. 2022).

Construction began on April 5, 2020. The hospital was opened to the Rio de Janeiro State Regulation Center (Central de Regulação do Estado do Rio de Janeiro) on May 19, 2020, gradually receiving its first patients for hospitalization. The hospital was concluded in the month in which the pandemic reached its peak and, precisely because of the elevated infection rate, expectations for the hospital’s inauguration and functioning were high. In addition, circulating news reported on the delay in delivering the field hospitals, an urgent measure to contain the growing number of deaths recorded daily in the country. A report published in May 2020 drew attention to the fact that, of the 1,300 beds promised by the field hospitals, only 200 of them, i.e. those made available by Fiocruz, had been delivered and were in operation (Centro hospitalar..., 20 maio 2020).

The hospital care and research unit was built using “clean room” technology and with characteristics that set it apart from the field units built around the country at the time. While most of the hospital projects were structured for temporary operation, the so-called field hospitals, Fiocruz’s Covid-19 Hospital Center was designed from the outset with the commitment to remain open after the pandemic and implemented as a legacy of Fiocruz for the Unified Health System (Sistema Único de Saúde, SUS) (Fiocruz, 19 maio 2020; Azevedo, 4 out. 2020).

Although having the peculiarity of aiming to be a permanent hospital, when comparing Fiocruz’s one to the other temporary hospitals designed during the covid-19 pandemic in the country, there are indisputable similarities, justified by the speed with which the project was carried out. In Brazil, most of the temporary hospitals were built using a tensile system, considered the most typical when dealing with ephemeral emergency architecture. This model is characterized by a structure “made up of rigid metal pieces, wood or even plastic with rapid assemblage and a flexible system,” common at events and fairs, “adapted for this purpose in a labyrinthine tangle of flame-retardant canvas tents, partitions, and elevated floors” (Ghisleni, 31 out. 2021).

The question of adaptability of space and materials used was central to the creation of temporary hospitals thought as a response to the sanitary crisis and, in addition to this point, the concern with the efficiency in containing virus transmissions in interior spaces (Ruprecht, 1 abr. 2020).

Another important aspect of these temporary constructions was the fact that they were intended to relieve the health system, bringing patients who were less affected by the disease. According to Fabio Racy, a specialist in disaster medicine and medical coordinator of the Pacaembu field hospital, managed by Hospital Israelita Albert Einstein, the covid-19 pandemic demanded many hospitalizations, saturating the health system and, therefore, “the field hospitals help mainly to relieve the need for beds for low-complexity covid-19 patients” (cited in Ruprecht, 1 abr. 2020).

The new hospital facility at Manguinhos campus was adapted to meet the most up-to-date health regulations and a construction process similar, to a certain extent, to the model of temporary hospital architecture in place at the same time it was built, casting doubts about its extended life span. Its construction in the emergency context provided it with the elements of a typical field hospital – modulation, rapid assemblage, and standardized installations.

The similarity with hospitals built for the covid-19 pandemic, including examples in other countries,^7^ and with the parameters prescribed by the World Health Organization (WHO) itself for the construction of Severe Acute Respiratory Infection (SARI) treatment units, does not hide the option adopted in the parameters of the project designed by Fiocruz for the new hospital facility. According to the premises in the guidelines presented at the course offered by the WHO for architects and engineers: “The SARI Facilities training package has been developed to meet the operational needs emerging with the covid-19 pandemic” (WHO, s.d.). In fact, by analyzing the project we can note that the new hospital built in Manguinhos followed the WHO course premises, confirming its emergency nature and raising doubts about its permanence without a high investment in structural maintenance.

The Covid-19 Hospital Center was completed with a total of 195 beds, all in intensive and semi-intensive single rooms with patient isolation. The project brings together the most modern technology and structure. All the beds operate with a negative air pressure isolation system, specifically for aerosol infections. Inside the individual rooms, a duct is responsible for sucking the contaminated air, which passes through a filtering system before being eliminated by chimneys installed on the outside of the building.

The hospital complex counts with its own sewage treatment center, conceived to treat coronavirus waste and ensure the safe disposal of the effluent generated. All the beds have high-efficiency care and are equipped with their own diagnostic support system, which includes x-ray, ultrasound, echocardiogram, computerized tomography machines, bronchoscopy and endoscopy services, clinical pathology, and transfusion center. The unit is self-sufficient – it has its own power supply, generators, water tanks, and all the infrastructure required for a hospital of this size, being independent from the other Fiocruz areas on campus. The hospital also has an exclusive entrance for ambulances and a helipad, as well as a technical support area with a pharmacy, nutrition, and a Sterile Material Center. In terms of administrative support, it has space for management, reception, and family assistance areas, and a call center. As for logistical support, the hospital is equipped with a security room, hospitality area, cafeteria, warehouse, and morgue.

From the patient care and research points of view, the covid-19 Hospital Center presents a characteristic that connects different temporalities in institutional history. The hospital is part of the Evandro Chagas National Institute of Infectious Diseases (Instituto Nacional de Infectologia Evandro Chagas, INI) and has maintained the link between clinical and therapeutic research and patient care since the beginning of the twentieth century (Santos, 2019). The new care and research facility was considered a key element in accelerating INI’s studies with the entire Fiocruz collaboration network throughout Brazil and internationally. According to INI’s Director Valdileia Veloso, one example is the WHO’s Solidarity clinical trial, investigating the drugs used to treat covid-19 (WHO, 2021).

The above-mentioned study investigates the effectiveness of four different drugs in treating the new coronavirus. In addition to the INI hospital center, 17 other hospitals in 12 Brazilian states participated in the study. “Coordinated in Brazil by Fiocruz, Solidarity is a randomized and adaptive clinical trial, which allows the therapeutic proposals to be altered as new scientific evidence emerges throughout the study” (Fiocruz, 2020). These relationships show how the institution has been present, aligned in a global effort, to provide quick responses on which drugs are effective in the treatment of covid-19 and which are ineffective and should not be used. They also demonstrate the institutional commitment on behalf of public health, which is clear in the speech of the then president of the foundation, Nísia Trindade Lima, during a site visit for the hospital inauguration, in which she praises Fiocruz’s role in battling the disease, the legacy of the new hospital, and its important contribution to the Brazilian Unified Health System:

At this time, when we are following with such concern the increase in cases and deaths in our country, and particularly in Rio de Janeiro, it is with great emotion that we hand over this hospital dedicated exclusively to covid-19 and which will remain a legacy for the Unified Health System (Sistema Único de Saúde, SUS). Fiocruz will continue to work ceaselessly to strengthen the actions of the SUS amid this humanitarian crisis that has had such a huge impact on the lives of the population and that brings so many challenges to a continental and unequal country like Brazil (Fiocruz, 19 maio 2020).^8^

## Discourses of modernity and permanence in the Manguinhos hospitals

The constructions of these two hospitals, separated by more than 100 years, allow us to analyze the strategies and the context in which each was conceived, from the point of view of the history of sciences and the point of view of the history of hospital architecture. Both hospitals were built within an institutional policy of combining treatment with clinical and therapeutic research, thus expanding a more attentive and permanent look at the issue, avoiding a merely emergency attitude to dealing with pandemics. Both hospitals were surrounded by the most modern technologies available at the time. However, their architectures reveal specific attitudes towards design.

The first hospital was conceived as part of a more ambitious plan, which had to be adapted due to situations unrelated to its development, such as the premature death of its designer. The result of a process in which different conceptions and formal structures were experimented with, involving at least two or three projects, the then Oswaldo Cruz Hospital pursued the typology of an isolation and pavilion hospital, inspired by structures developed from the studies of Florence Nightingale and Louis Pasteur, as we have seen. According to Nightingale (1863, p.56):

The first principle of hospital construction is to divide the sick among separate pavilions. By a hospital pavilion is meant a detached block of building, capable of containing the largest number of beds that can be placed safely in it, together with suitable nurses’ rooms, ward sculleries, lavatories, baths, water-closets, all complete, proportioned to the number of sick, and quite unconnected with any other pavilions of which the hospital may consist, or with the general administrative offices, except by light airy passages or corridors. A pavilion is indeed a separate detached hospital, which has, or ought to have, as little connexion in its ventilation with any other part of the hospital, as if it were really a separate establishment miles away.

To this end, the hospital was structured around the first floor, a basement, and an attic. On the main floor, it distributed wards at the ends of the space, interspersing them with places for reception and care, and for shelter for the doctors and nurses. Both the basement and the attic revealed themselves as support areas and technical space, for the installation of elements to contribute to aerate the wards. In the same way, the external balconies reduce the incidence of the sun and help to mitigate the strong heat of the tropics. Its location obeyed the need for distance and isolation from the other component buildings of the institution, as well as from other areas of the city of Rio de Janeiro itself. Operational support facilities were built around it, such as a laundry, kitchen, vivarium, and outpatient clinics.

Its permanence on campus, more than 100 years after it was built and despite some attempts to emptying and replacing it, reveals the hospital’s historical and heritage significance, and therefore its interest in preservation. The arrival of the new hospital, built amidst the covid-19 pandemic, alerted us to its continuation as a hospital and its subsequent safekeeping.

The modern health hospital complex of the former IOC came about through the construction of one of the six pavilions Oswaldo Cruz envisioned (Benchimol, 1990), as we have seen. This building, which was conceived as part of this large structure for treating patients separated by disease in different pavilions, centralized all the treatment and research activities for the most diverse illnesses, and thus, right from the start, exceeded the use and occupation for which it was built. It is therefore clear that the old hospital has been undergoing adaptations since the beginning of its existence, either to occupy the spaces or to modernize the facilities, to ensure compliance with regulations, better patient care, and research.

The issue of preserving the old hospital building because of preserving its original use has been much discussed, given the architectural features that identify it as an important health heritage site. The analyses that have been carried out over the years certify that the building’s volumetry has been preserved. However, some important aspects have been lost in the internal spaces, such as the beds’ partitions, installations, and the original air exchange and air conditioning system, as well as in the external ambiance and the constructive elements, especially the roof tiling, balcony railings, window frames, and accesses, among others. These losses have almost irreversibly compromised some of the most important features of the pavilion architecture typology, such as the air renewal system the wards, and the distance from other buildings.

On the other hand, as the Oswaldo Cruz Foundation’s first hospital, there is an appeal regarding its original use and the fact that losing this status would reduce its value as a monument. However, if this impossibility of preservation is true, is its original use the most significant value to be discussed? Given the losses, the difficulties of adaptation projects, installations, and even simple conservation services, it is necessary to analyze the issue more closely, considering that although the original use is important, the building itself is even more significant and relevant, because it is only with its material characteristics preserved that it is possible to add value and recognize its antiquity.

The original use is a special quality of this historic building, recognized as one of its values, which were attributed in the document Preservation and Management Policy of Cultural Collections in Science and Health (*Política de preservação e gestão de acervos culturais das ciências e da saúde da Casa de Oswaldo Cruz*) (Fiocruz, 2013), the Fiocruz unit responsible for the institution’s preservation. Now, the historic hospital is in the process of being recognized as a heritage site by the National Historical and Artistic Heritage Institute (Instituto de Patrimônio Histórico e Artístico Nacional, IPHAN). In recent years, IPHAN experts have visited the building to analyze and verify whether the changes it has undergone have altered its character-defining features, to the point of compromising its heritage protection by the agency: “A future recognition from IPHAN will be an important step to add to the advocacy actions of this health heritage” (Costa, Martire, 2023, p.70).

From an exclusively architectural point of view, there is no expectation that the hospital’s original attributes will be preserved, due to the necessary and recurring adaptations. And even though the hospital is considered an architectural heritage building, it is necessary to look at it from a more contemporary point of view, since its main function is to care for sick people and carry out research.

At this point, the construction of a new hospital under INI’s responsibility will help the teams responsible for the old building to adapt the historic architecture to the contemporary nature of its activities as a hospital, with the challenge of respecting its preservation. It is important to keep in mind that this change of clinical activities in the new hospital may bring new meanings to the old one, but it must not give way to its abandonment. Careful analysis should then turn to the heritage question, aiming for a reuse that revalues the architecture and favors the preservation of the architecture of the old hospital from the point of view of remembering the history of health.

In turn, the new hospital facilities represented an opportunity for Fiocruz to have a hospital that met the latest health standards, although the construction process of the new hospital did not allow for a very long lifetime, according to the architect who accompanied the work on site. We know that it was built in an emergency context and adopted elements of a typical field hospital, namely modulation, rapid assembly, and standardized installations.

INI does not recognize that the new hospital complex has an operation expiration date, another characteristic of field hospitals. According to a report on May 20, 2020, aired on the midday edition of *Jornal Hoje* (TV Globo), the institution stated that “the Covid-19 Hospital Center is not a field hospital and that ‘it will remain standing after the pandemic’” (Centro hospitalar..., 20 maio 2020). The architect Luiz Carlos Toledo (29 jun. 2020), in his article on the hospitals built as part of the fight against covid-19 in Rio de Janeiro, also went so far as to say that, in the case of Fiocruz, its hospital would have been built as “permanent, integrated into the Unified Health System.” The architect draws attention to the mistake of building field hospitals for this purpose, because, according to him, “they will still have to function for a long time,” due to the permanence of the pandemic, and because the structure of such hospitals would not be the most appropriate for the situation.

In addition, Toledo (29 jun. 2020) recognizes that the example set by the new Fiocruz hospital has drawn attention to some important aspects for the hospital in the future: “195 intensive and semi-intensive care beds, assisted by an exhaust and air filtration system to reduce the risk of transmission of covid-19 in the hospital environment itself, protecting health teams and patients from greater exposure to the virus.” Other aspects could be improved, such as the creation of individual Intensive Care Units, more efficient internal air exchange systems, more space for beds, and more attention to patients, with improved communication technologies between doctors, patients, and their families, as well as the recovery of traditional elements of old hospitals, such as natural ventilation and insolation mechanisms, so neglected in contemporary hospitals.

As we have seen, the proposals for the two hospitals, both built for emergencies and separated by more than 100 years of construction, reveal different design attitudes and, consequently, require different forms of preservation. The new hospital resembles the historic one in that it was built in the face of a strong call for answers in the scientific field to shape disease and contain its advance. This time, the argument centered on the covid-19 pandemic, motivating studies and research on an international scale to shape the clinical, therapeutic, and prophylactic framework of the disease.

## Final considerations

From the perspective of the history of health and disease, the trigger for the materialization of the old and new hospitals was the epidemics of Chagas disease and covid-19.^9^ Despite their historical peculiarities, etiology, degree of contamination and lethality, and the entire socio-cultural context surrounding the formation of the diseases, both were on the political agenda of the country and of the institution that is now Fiocruz. The history of these diseases involved contexts of scientific concern at times of doubt about how to proceed when facing new illnesses. There were moments of uncertainty about the signs and evolution of clinical conditions, and social fear about the growing number of deaths. In this context, scientific answers circulated through these hospitals and contributed, within a broad network of studies, to defining, containing, treating, and preventing the outbreak of infections, both at the beginning of the twentieth century and recently, at the beginning of the twenty-first century. At both times, research was directly aligned with studies carried out on a global scale and had a space for hospital experimentation in Manguinhos.

The old hospital, the centenary Oswaldo Cruz Hospital, imposes the need to constantly reflect on the preservation of its space and its memory. The new hospital, the Covid-19 Hospital Center, presents us with a problem of “our own time.”^10^ Its interpretation exposes latent issues of the present time, presenting the difficulty of experiencing and studying historical facts and contemporaneity at the same time.

However, as an object of analysis, it broadens debates surrounding both the health crisis and the challenges of keeping the new hospital in this institution. The issues raised by both hospitals make it possible to keep up to date with possible interpretations and testimonies of how health policies are made and how medical care is conducted. In both cases, research, clinical, and therapeutic protocols were developed that proved crucial in dealing with the illnesses of their respective eras.

Preserving or analyzing their constructive forms and the contexts in which they were both built helps the institution to think of strategies for their maintenance, whether from a heritage perspective and for valuing their memory, or because of the importance of the necessary assistance, no longer because of the urgency of the pandemic, but because of the need to continue studies on diseases, such as covid-19, and because of the lack of hospitals in the city and the region. In this sense, the paper presented here seeks to provide reflections that can contribute to a better understanding of both hospitals and thus integrate studies on the history of science and health.

## NOTES

1 This paper presents part of the reflections that emerged from the research “Places for science: evolution of the Fiocruz campuses in Manguinhos and Mata Atlântica” (“Lugares para a ciência: evolução dos *campi* da Fiocruz em Manguinhos e Mata Atlântica”), funded by Oswaldo Cruz/Fiocruz’s Institutional Development Incentive Program (Programa de Incentivo ao Desenvolvimento Institucional, PIDI) of Casa de Oswaldo Cruz/Fiocruz.

2 For the institution’s history, see Aragão (1950); Stepan (1976); Benchimol (1990); Benchimol e Teixeira (1993); Cukierman (2007).

3 For hospital design and construction, see Benchimol (1990); Santos (2019).

4 The regulations approved for the Oswaldo Cruz Institute (Instituto Oswaldo Cruz, IOC) on March 19, 1908, sanctioned the sale of biological products, such as sera and vaccines. This prerogative was responsible for “its ability to survive the shortage of budgetary resources voted by Congress, under more favorable conditions than other public establishments in the country” (Benchimol, 1990, p.38).

5 An analysis of hospital architecture can be found in Benchimol (1990, v.2, p.39-54); Costa (2011); and Santos (2019). On hospital architecture in the first decades of the twentieth century in the city of Rio de Janeiro, see Costa (2011); Sanglard (2008); Porto (2008).

6 Process n.25389.000027/2020-78 and bidding contract n.11/2020.

7 For information on other covid-19 pandemic hospitals built around the world, see Bittencourt, Vilas-Boas, Silva (2021).

8 In this and others citations of texts from non-English languages, a free translation has been provided.

9 In the paper, we treat diseases not only as biological and physical entities but as social and cultural phenomena, as problematized by Cunningham; Williams (1992); Rosenberg, Golden (1997).

10 The history of the present time, as a field of study, leads us to reflect on the writing of history concomitant with our existence. It brings us closer to the limits and challenges of narrative practice situated in “our own time,” a concept explored by historiography in the last two decades. On present time, see Dosse (2009); Ferreira (2018, 2000).
